# Benzyl *N*-(4-pyrid­yl)carbamate

**DOI:** 10.1107/S160053681000098X

**Published:** 2010-01-20

**Authors:** Hua Fang, Mei-Juan Fang, Yi-Ping Zhang

**Affiliations:** a The Third Institute of Oceanography of the State Oceanic Administration, Xiamen 361005, People’s Republic of China; b Department of Pharmaceutical Science, Medical College, Xiamen University, Xiamen 361005, People’s Republic of China

## Abstract

The title compound, C_13_H_12_N_2_O_2_, was obtained by the reaction of 4-amino­pyridine and benzyl chloro­formate in tetra­hydro­furan. The crystal structure contains N—H⋯N hydrogen bonds between two unique mol­ecules within layers and anti­parallel C—O⋯O—C inter­actions [O⋯O = 3.06 (3) Å] between the two mol­ecules of the asymmetric unit.

## Related literature

The title compound was synthesized in order to investigate the nature of its reversion tetrodotoxin-induced cardiorespiratory depression, see: Chang *et al.* (1997 ▶). For a related structure, see: Zheng *et al.* (2005 ▶).
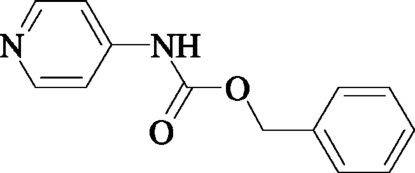


## Experimental

### 

#### Crystal data

C_13_H_12_N_2_O_2_
*M* *_r_* = 228.25
Monoclinic, 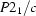

*a* = 11.9439 (5) Å
*b* = 13.2120 (6) Å
*c* = 14.6574 (7) Å
β = 98.418 (4)°
*V* = 2288.06 (18) Å^3^
*Z* = 8
Mo *K*α radiation
μ = 0.09 mm^−1^
*T* = 293 K
0.30 × 0.22 × 0.18 mm

#### Data collection

Bruker SMART APEX area-detector diffractometer
Absorption correction: multi-scan (*SADABS*; Bruker, 2001 ▶) *T*_min_ = 0.973, *T*_max_ = 0.984
11332 measured reflections
4028 independent reflections
2831 reflections with *I* > 2σ(*I*)
*R*_int_ = 0.039

#### Refinement

*R*[*F* ^2^ > 2σ(*F* ^2^)] = 0.036
*wR*(*F* ^2^) = 0.070
*S* = 1.05
4028 reflections
307 parameters
H-atom parameters constrained
Δρ_max_ = 0.17 e Å^−3^
Δρ_min_ = −0.17 e Å^−3^

### 

Data collection: *SMART* (Bruker, 2001 ▶); cell refinement: *SAINT* (Bruker, 2001 ▶); data reduction: *SAINT*; program(s) used to solve structure: *SHELXS97* (Sheldrick, 2008 ▶); program(s) used to refine structure: *SHELXL97* (Sheldrick, 2008 ▶); molecular graphics: *ORTEP-3* (Farrugia, 1997 ▶) and *PLATON* (Spek, 2009 ▶); software used to prepare material for publication: *SHELXL97*.

## Supplementary Material

Crystal structure: contains datablocks I, global. DOI: 10.1107/S160053681000098X/jh2125sup1.cif

Structure factors: contains datablocks I. DOI: 10.1107/S160053681000098X/jh2125Isup2.hkl

Additional supplementary materials:  crystallographic information; 3D view; checkCIF report

## Figures and Tables

**Table 1:** Hydrogen-bond geometry (Å, °)

*D*—H⋯*A*	*D*—H	H⋯*A*	*D*⋯*A*	*D*—H⋯*A*
N2′—H2′*C*⋯N1^i^	0.86	2.09	2.9460 (16)	171
N2—H2*C*⋯N1′^ii^	0.86	2.11	2.9630 (18)	170

Symmetry codes: (i) 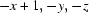; (ii) 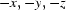.

**Fig. 1.**
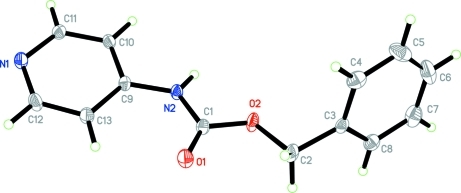
ORTEPII (Farrugia, 1997) plot of the title molecule with displacement ellipsoids srawn at the 30% probability level. H atoms are drawn as spheres of arbitrary radii.

**Fig. 2.**
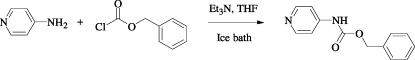
The formation of the title compound.

## supplementary crystallographic information

### Comment

The title compound, (I), a 4-aminopyridine analog, was synthesized for investigation of the nature of its reversion tetrodotoxin-induced cardiorespiratorydepression (Chang *et al.*,  1997). The crystal packing is stabilized by stronger N-H···N hydrogen bonds between molecules within layers and antiparallel C-O ··· O-C interactions between the two molecules of the asymmetric unit, and bond lengths and angles are in agreement with values reported for a similar compound (Zheng *et al.*, 2005). The dihedral angle between the planes of the phenzene ring and pyridine ring systems is 66.2 (3) °.

### Experimental

A solution of dry tetrahydrofuran (15 ml) containing 4-aminopyridine (5 mmol, 0.47 g) and triethylamine (0.70 ml) was added dropwise to the solution of tetrahydrofuran (15 ml) containing the benzyl chloroformate (5 mmol, 0.85 g). The reaction mixture was stirred for 6 h in ice bath and the solvent was then removed under reduced pressure to give a residue, which was extracted with ethyl acetate (3 × 15 ml). The solution was dried over anhydrous MgSO_4_ and concentrated under vacuum to obtain a slurry residue, which was purified by silica gel column chromatography (petroleum ether/ethyl acetate = 2:1) to give products I as colorless amorphous solids. Single crystals of (I) suitable for X-ray analysis were obtained by slow evaporation of a petroleum ether /dichloromethane solution (1:1 *v*/*v*).

### Refinement

All H atoms were placed in geometrically idealized positions and treated as riding on their parent atoms, with C—H = 0.93 (aromatic), 0.97 (CH_2_), N—H = 0.86 Å and *U*_iso_(H) = 1.2*U*_eq_ (aromatic C, CH_2_ and N).

### Crystal data

**Table:**

C_13_H_12_N_2_O_2_	*F*(000) = 960
*M**_r_* = 228.25	*D*_x_ = 1.325 Mg m^−^^3^
Monoclinic, *P*2_1_/*c*	Mo *K*α radiation, λ = 0.71073 Å
Hall symbol: -P 2ybc	Cell parameters from 2895 reflections
*a* = 11.9439 (5) Å	θ = 2.1–26.4°
*b* = 13.2120 (6) Å	µ = 0.09 mm^−^^1^
*c* = 14.6574 (7) Å	*T* = 293 K
β = 98.418 (4)°	Chunk, colorless
*V* = 2288.06 (18) Å^3^	0.30 × 0.22 × 0.18 mm
*Z* = 8	

### Data collection

**Table:**

Bruker APEX area-detector diffractometer	4028 independent reflections
Radiation source: fine-focus sealed tube	2831 reflections with *I* > 2σ(*I*)
graphite	*R*_int_ = 0.039
φ and ω scan	θ_max_ = 25.0°, θ_min_ = 2.3°
Absorption correction: multi-scan (*SADABS*; Bruker, 2001)	*h* = −14→12
*T*_min_ = 0.973, *T*_max_ = 0.984	*k* = −15→15
11332 measured reflections	*l* = −17→17

### Refinement

**Table:**

Refinement on *F*^2^	Primary atom site location: structure-invariant direct methods
Least-squares matrix: full	Secondary atom site location: difference Fourier map
*R*[*F*^2^ > 2σ(*F*^2^)] = 0.036	Hydrogen site location: inferred from neighbouring sites
*wR*(*F*^2^) = 0.070	H-atom parameters constrained
*S* = 1.05	*w* = 1/[σ^2^(*F*_o_^2^) + (0.0229*P*)^2^ + 0.110*P*] where *P* = (*F*_o_^2^ + 2*F*_c_^2^)/3
4028 reflections	(Δ/σ)_max_ < 0.001
307 parameters	Δρ_max_ = 0.17 e Å^−^^3^
0 restraints	Δρ_min_ = −0.17 e Å^−^^3^

### Special details

**Table:**

Geometry. All e.s.d.'s (except the e.s.d. in the dihedral angle between two l.s. planes) are estimated using the full covariance matrix. The cell e.s.d.'s are taken into account individually in the estimation of e.s.d.'s in distances, angles and torsion angles; correlations between e.s.d.'s in cell parameters are only used when they are defined by crystal symmetry. An approximate (isotropic) treatment of cell e.s.d.'s is used for estimating e.s.d.'s involving l.s. planes.
Refinement. Refinement of *F*^2^ against ALL reflections. The weighted *R*-factor *wR* and goodness of fit *S* are based on *F*^2^, conventional *R*-factors *R* are based on *F*, with *F* set to zero for negative *F*^2^. The threshold expression of *F*^2^ > σ(*F*^2^) is used only for calculating *R*-factors(gt) *etc*. and is not relevant to the choice of reflections for refinement. *R*-factors based on *F*^2^ are statistically about twice as large as those based on *F*, and *R*- factors based on ALL data will be even larger.

### Fractional atomic coordinates and isotropic or equivalent isotropic displacement parameters (Å^2^)

**Table:**

	*x*	*y*	*z*	*U*_iso_*/*U*_eq_	
N2'	0.16150 (9)	0.18181 (9)	−0.00328 (9)	0.0227 (3)	
H2'C	0.2140	0.1458	−0.0216	0.027*	
O1	0.42587 (8)	0.17805 (9)	0.26686 (8)	0.0335 (3)	
O2	0.24197 (8)	0.13329 (8)	0.22775 (8)	0.0330 (3)	
O1'	0.13791 (8)	0.32072 (8)	0.08678 (8)	0.0301 (3)	
O2'	0.30953 (8)	0.27290 (8)	0.05366 (8)	0.0288 (3)	
N2	0.36888 (9)	0.04846 (9)	0.16579 (9)	0.0240 (3)	
H2C	0.3088	0.0179	0.1403	0.029*	
C1	0.35408 (12)	0.12531 (12)	0.22453 (11)	0.0241 (4)	
N1'	−0.16725 (10)	0.07480 (10)	−0.09588 (10)	0.0297 (3)	
C9	0.47128 (11)	0.01402 (12)	0.14254 (10)	0.0217 (4)	
C3'	0.48132 (13)	0.36576 (11)	0.08454 (12)	0.0269 (4)	
C13	0.57368 (12)	0.06602 (13)	0.16021 (12)	0.0292 (4)	
H13A	0.5789	0.1278	0.1909	0.035*	
C10	0.47072 (12)	−0.07770 (12)	0.09719 (11)	0.0254 (4)	
H10A	0.4040	−0.1147	0.0844	0.031*	
C1'	0.19585 (12)	0.26347 (12)	0.04966 (11)	0.0229 (4)	
C9'	0.05051 (12)	0.14969 (11)	−0.03138 (11)	0.0200 (4)	
C10'	0.03345 (12)	0.07278 (12)	−0.09590 (11)	0.0244 (4)	
H10B	0.0948	0.0447	−0.1193	0.029*	
C13'	−0.04470 (12)	0.18983 (12)	0.00023 (11)	0.0257 (4)	
H13B	−0.0382	0.2421	0.0432	0.031*	
N1	0.66870 (10)	−0.06576 (10)	0.08657 (9)	0.0290 (3)	
C11'	−0.07391 (12)	0.03782 (12)	−0.12545 (12)	0.0281 (4)	
H11A	−0.0827	−0.0144	−0.1684	0.034*	
C3	0.09174 (12)	0.16792 (12)	0.31022 (11)	0.0243 (4)	
C11	0.56904 (12)	−0.11411 (12)	0.07098 (12)	0.0283 (4)	
H11B	0.5662	−0.1760	0.0406	0.034*	
C2'	0.36095 (12)	0.36077 (12)	0.10249 (12)	0.0333 (4)	
H2'A	0.3585	0.3543	0.1681	0.040*	
H2'B	0.3206	0.4218	0.0804	0.040*	
C12	0.66733 (12)	0.02252 (13)	0.13053 (12)	0.0328 (4)	
H12A	0.7352	0.0579	0.1422	0.039*	
C8	−0.00463 (13)	0.21433 (13)	0.26577 (12)	0.0320 (4)	
H8A	0.0015	0.2712	0.2294	0.038*	
C4	0.08082 (15)	0.08481 (13)	0.36471 (12)	0.0367 (4)	
H4A	0.1454	0.0542	0.3961	0.044*	
C2	0.20694 (13)	0.20348 (13)	0.29487 (12)	0.0348 (5)	
H2A	0.2034	0.2721	0.2711	0.042*	
H2B	0.2594	0.2017	0.3520	0.042*	
C4'	0.50592 (14)	0.36917 (12)	−0.00500 (13)	0.0351 (4)	
H4'A	0.4472	0.3691	−0.0543	0.042*	
C8'	0.56941 (14)	0.36833 (13)	0.15684 (13)	0.0388 (5)	
H8'A	0.5545	0.3653	0.2173	0.047*	
C12'	−0.14897 (12)	0.14942 (12)	−0.03432 (12)	0.0309 (4)	
H12B	−0.2119	0.1767	−0.0127	0.037*	
C5'	0.61669 (16)	0.37268 (13)	−0.02173 (15)	0.0465 (5)	
H5'A	0.6322	0.3726	−0.0821	0.056*	
C6	−0.12000 (16)	0.09262 (18)	0.32858 (15)	0.0521 (6)	
H6A	−0.1911	0.0671	0.3345	0.063*	
C7	−0.11059 (14)	0.17636 (16)	0.27524 (14)	0.0453 (5)	
H7A	−0.1754	0.2079	0.2453	0.054*	
C7'	0.68094 (15)	0.37554 (14)	0.13934 (17)	0.0522 (6)	
H7'A	0.7400	0.3799	0.1882	0.063*	
C6'	0.70368 (16)	0.37628 (14)	0.05031 (18)	0.0524 (6)	
H6'A	0.7782	0.3792	0.0388	0.063*	
C5	−0.02418 (18)	0.04609 (15)	0.37357 (14)	0.0495 (6)	
H5A	−0.0304	−0.0110	0.4096	0.059*	

### Atomic displacement parameters (Å^2^)

**Table:**

	*U*^11^	*U*^22^	*U*^33^	*U*^12^	*U*^13^	*U*^23^
N2'	0.0176 (7)	0.0229 (7)	0.0278 (8)	0.0018 (5)	0.0046 (6)	−0.0046 (7)
O1	0.0266 (6)	0.0403 (7)	0.0339 (7)	−0.0061 (5)	0.0051 (6)	−0.0154 (6)
O2	0.0215 (6)	0.0421 (7)	0.0365 (7)	0.0007 (5)	0.0081 (5)	−0.0205 (6)
O1'	0.0277 (6)	0.0280 (7)	0.0356 (7)	0.0020 (5)	0.0078 (5)	−0.0092 (6)
O2'	0.0214 (6)	0.0273 (7)	0.0383 (7)	−0.0062 (4)	0.0065 (5)	−0.0115 (6)
N2	0.0162 (7)	0.0282 (8)	0.0276 (8)	−0.0019 (5)	0.0028 (6)	−0.0090 (7)
C1	0.0233 (9)	0.0283 (10)	0.0212 (9)	0.0012 (7)	0.0051 (7)	−0.0013 (8)
N1'	0.0224 (7)	0.0310 (9)	0.0357 (9)	−0.0012 (6)	0.0043 (6)	−0.0044 (7)
C9	0.0185 (8)	0.0276 (10)	0.0191 (9)	0.0019 (6)	0.0030 (7)	0.0019 (8)
C3'	0.0293 (9)	0.0198 (9)	0.0318 (11)	−0.0058 (7)	0.0054 (8)	−0.0060 (8)
C13	0.0241 (9)	0.0307 (10)	0.0330 (11)	−0.0027 (7)	0.0048 (8)	−0.0103 (9)
C10	0.0205 (8)	0.0269 (10)	0.0295 (10)	−0.0034 (7)	0.0055 (7)	−0.0029 (8)
C1'	0.0242 (9)	0.0223 (9)	0.0221 (9)	−0.0004 (7)	0.0033 (7)	0.0016 (8)
C9'	0.0192 (8)	0.0202 (9)	0.0206 (9)	−0.0012 (6)	0.0028 (7)	0.0039 (7)
C10'	0.0203 (8)	0.0265 (9)	0.0271 (10)	0.0009 (7)	0.0061 (7)	−0.0019 (8)
C13'	0.0239 (9)	0.0262 (10)	0.0271 (10)	0.0012 (7)	0.0044 (7)	−0.0036 (8)
N1	0.0222 (7)	0.0331 (9)	0.0317 (9)	0.0007 (6)	0.0047 (6)	−0.0040 (7)
C11'	0.0271 (9)	0.0271 (10)	0.0305 (10)	−0.0019 (7)	0.0055 (8)	−0.0047 (8)
C3	0.0266 (9)	0.0256 (9)	0.0213 (10)	0.0015 (7)	0.0056 (7)	−0.0087 (8)
C11	0.0258 (9)	0.0263 (10)	0.0331 (11)	0.0001 (7)	0.0055 (8)	−0.0047 (8)
C2'	0.0333 (10)	0.0257 (10)	0.0416 (12)	−0.0096 (7)	0.0083 (9)	−0.0127 (9)
C12	0.0205 (9)	0.0409 (12)	0.0370 (11)	−0.0067 (7)	0.0044 (8)	−0.0096 (9)
C8	0.0373 (10)	0.0330 (11)	0.0258 (10)	0.0034 (8)	0.0051 (8)	−0.0031 (8)
C4	0.0448 (11)	0.0343 (11)	0.0298 (11)	0.0040 (8)	0.0019 (9)	−0.0028 (9)
C2	0.0327 (10)	0.0393 (11)	0.0347 (11)	−0.0014 (8)	0.0120 (8)	−0.0194 (9)
C4'	0.0386 (10)	0.0310 (10)	0.0357 (11)	−0.0110 (8)	0.0058 (9)	−0.0024 (9)
C8'	0.0431 (11)	0.0353 (11)	0.0357 (12)	−0.0068 (8)	−0.0021 (9)	−0.0078 (9)
C12'	0.0213 (9)	0.0340 (11)	0.0387 (11)	0.0033 (7)	0.0088 (8)	−0.0030 (9)
C5'	0.0514 (13)	0.0368 (12)	0.0574 (14)	−0.0174 (9)	0.0287 (11)	−0.0123 (10)
C6	0.0404 (12)	0.0747 (16)	0.0449 (14)	−0.0236 (11)	0.0189 (11)	−0.0234 (12)
C7	0.0291 (10)	0.0607 (14)	0.0442 (13)	0.0035 (9)	−0.0009 (9)	−0.0149 (12)
C7'	0.0303 (11)	0.0444 (13)	0.0755 (17)	−0.0052 (8)	−0.0141 (11)	−0.0119 (12)
C6'	0.0327 (11)	0.0376 (12)	0.090 (2)	−0.0091 (9)	0.0212 (13)	−0.0203 (12)
C5	0.0732 (15)	0.0442 (13)	0.0340 (12)	−0.0213 (11)	0.0174 (11)	−0.0027 (10)

### Geometric parameters (Å, °)

**Table:**

N2'—C1'	1.3569 (19)	N1—C11	1.3407 (19)
N2'—C9'	1.3954 (17)	C11'—H11A	0.9300
N2'—H2'C	0.8600	C3—C4	1.375 (2)
O1—C1	1.2042 (18)	C3—C8	1.380 (2)
O2—C1	1.3509 (16)	C3—C2	1.502 (2)
O2—C2	1.4572 (17)	C11—H11B	0.9300
O1'—C1'	1.2072 (17)	C2'—H2'A	0.9700
O2'—C1'	1.3561 (16)	C2'—H2'B	0.9700
O2'—C2'	1.4520 (17)	C12—H12A	0.9300
N2—C1	1.3595 (19)	C8—C7	1.387 (2)
N2—C9	1.3932 (17)	C8—H8A	0.9300
N2—H2C	0.8600	C4—C5	1.378 (2)
N1'—C12'	1.333 (2)	C4—H4A	0.9300
N1'—C11'	1.3457 (18)	C2—H2A	0.9700
C9—C10	1.382 (2)	C2—H2B	0.9700
C9—C13	1.394 (2)	C4'—C5'	1.381 (2)
C3'—C4'	1.387 (2)	C4'—H4'A	0.9300
C3'—C8'	1.380 (2)	C8'—C7'	1.396 (2)
C3'—C2'	1.500 (2)	C8'—H8'A	0.9300
C13—C12	1.383 (2)	C12'—H12B	0.9300
C13—H13A	0.9300	C5'—C6'	1.369 (3)
C10—C11	1.3749 (19)	C5'—H5'A	0.9300
C10—H10A	0.9300	C6—C7	1.369 (3)
C9'—C10'	1.383 (2)	C6—C5	1.379 (3)
C9'—C13'	1.3940 (19)	C6—H6A	0.9300
C10'—C11'	1.372 (2)	C7—H7A	0.9300
C10'—H10B	0.9300	C7'—C6'	1.371 (3)
C13'—C12'	1.381 (2)	C7'—H7'A	0.9300
C13'—H13B	0.9300	C6'—H6'A	0.9300
N1—C12	1.334 (2)	C5—H5A	0.9300
			
C1'—N2'—C9'	127.25 (12)	O2'—C2'—H2'A	110.3
C1'—N2'—H2'C	116.4	C3'—C2'—H2'A	110.3
C9'—N2'—H2'C	116.4	O2'—C2'—H2'B	110.3
C1—O2—C2	117.22 (12)	C3'—C2'—H2'B	110.3
C1'—O2'—C2'	116.32 (11)	H2'A—C2'—H2'B	108.5
C1—N2—C9	126.80 (13)	N1—C12—C13	125.66 (14)
C1—N2—H2C	116.6	N1—C12—H12A	117.2
C9—N2—H2C	116.6	C13—C12—H12A	117.2
O1—C1—O2	124.86 (14)	C7—C8—C3	120.14 (17)
O1—C1—N2	127.57 (13)	C7—C8—H8A	119.9
O2—C1—N2	107.58 (13)	C3—C8—H8A	119.9
C12'—N1'—C11'	115.17 (13)	C3—C4—C5	121.07 (18)
C10—C9—N2	117.48 (13)	C3—C4—H4A	119.5
C10—C9—C13	117.66 (13)	C5—C4—H4A	119.5
N2—C9—C13	124.85 (14)	O2—C2—C3	105.19 (12)
C4'—C3'—C8'	118.84 (15)	O2—C2—H2A	110.7
C4'—C3'—C2'	120.56 (16)	C3—C2—H2A	110.7
C8'—C3'—C2'	120.59 (15)	O2—C2—H2B	110.7
C12—C13—C9	117.70 (15)	C3—C2—H2B	110.7
C12—C13—H13A	121.2	H2A—C2—H2B	108.8
C9—C13—H13A	121.2	C3'—C4'—C5'	120.68 (18)
C9—C10—C11	119.81 (14)	C3'—C4'—H4'A	119.7
C9—C10—H10A	120.1	C5'—C4'—H4'A	119.7
C11—C10—H10A	120.1	C3'—C8'—C7'	120.08 (18)
O1'—C1'—O2'	124.01 (14)	C3'—C8'—H8'A	120.0
O1'—C1'—N2'	127.63 (14)	C7'—C8'—H8'A	120.0
O2'—C1'—N2'	108.35 (12)	N1'—C12'—C13'	125.71 (14)
C10'—C9'—N2'	117.64 (12)	N1'—C12'—H12B	117.1
C10'—C9'—C13'	117.46 (14)	C13'—C12'—H12B	117.1
N2'—C9'—C13'	124.90 (14)	C6'—C5'—C4'	120.17 (19)
C9'—C10'—C11'	120.04 (13)	C6'—C5'—H5'A	119.9
C9'—C10'—H10B	120.0	C4'—C5'—H5'A	119.9
C11'—C10'—H10B	120.0	C7—C6—C5	120.11 (17)
C12'—C13'—C9'	117.82 (15)	C7—C6—H6A	119.9
C12'—C13'—H13B	121.1	C5—C6—H6A	119.9
C9'—C13'—H13B	121.1	C6—C7—C8	120.13 (18)
C12—N1—C11	115.19 (12)	C6—C7—H7A	119.9
N1'—C11'—C10'	123.78 (16)	C8—C7—H7A	119.9
N1'—C11'—H11A	118.1	C6'—C7'—C8'	120.2 (2)
C10'—C11'—H11A	118.1	C6'—C7'—H7'A	119.9
C4—C3—C8	119.03 (15)	C8'—C7'—H7'A	119.9
C4—C3—C2	120.24 (15)	C5'—C6'—C7'	119.99 (17)
C8—C3—C2	120.62 (16)	C5'—C6'—H6'A	120.0
N1—C11—C10	123.99 (15)	C7'—C6'—H6'A	120.0
N1—C11—H11B	118.0	C6—C5—C4	119.50 (19)
C10—C11—H11B	118.0	C6—C5—H5A	120.2
O2'—C2'—C3'	107.20 (12)	C4—C5—H5A	120.2
			
C2—O2—C1—O1	−7.0 (2)	C4'—C3'—C2'—O2'	56.18 (19)
C2—O2—C1—N2	173.09 (13)	C8'—C3'—C2'—O2'	−124.65 (17)
C9—N2—C1—O1	0.4 (3)	C11—N1—C12—C13	0.1 (3)
C9—N2—C1—O2	−179.66 (14)	C9—C13—C12—N1	−0.4 (3)
C1—N2—C9—C10	166.73 (15)	C4—C3—C8—C7	−0.9 (2)
C1—N2—C9—C13	−14.4 (2)	C2—C3—C8—C7	175.37 (15)
C10—C9—C13—C12	0.5 (2)	C8—C3—C4—C5	1.6 (2)
N2—C9—C13—C12	−178.37 (15)	C2—C3—C4—C5	−174.66 (15)
N2—C9—C10—C11	178.65 (15)	C1—O2—C2—C3	−157.99 (14)
C13—C9—C10—C11	−0.3 (2)	C4—C3—C2—O2	77.04 (18)
C2'—O2'—C1'—O1'	−3.0 (2)	C8—C3—C2—O2	−99.19 (16)
C2'—O2'—C1'—N2'	175.94 (13)	C8'—C3'—C4'—C5'	1.5 (2)
C9'—N2'—C1'—O1'	4.4 (3)	C2'—C3'—C4'—C5'	−179.31 (14)
C9'—N2'—C1'—O2'	−174.45 (13)	C4'—C3'—C8'—C7'	0.8 (3)
C1'—N2'—C9'—C10'	171.04 (15)	C2'—C3'—C8'—C7'	−178.39 (15)
C1'—N2'—C9'—C13'	−8.5 (2)	C11'—N1'—C12'—C13'	−0.2 (2)
N2'—C9'—C10'—C11'	179.55 (14)	C9'—C13'—C12'—N1'	−0.1 (2)
C13'—C9'—C10'—C11'	−0.9 (2)	C3'—C4'—C5'—C6'	−2.2 (3)
C10'—C9'—C13'—C12'	0.6 (2)	C5—C6—C7—C8	0.4 (3)
N2'—C9'—C13'—C12'	−179.87 (14)	C3—C8—C7—C6	−0.1 (3)
C12'—N1'—C11'—C10'	−0.1 (2)	C3'—C8'—C7'—C6'	−2.4 (3)
C9'—C10'—C11'—N1'	0.7 (2)	C4'—C5'—C6'—C7'	0.5 (3)
C12—N1—C11—C10	0.1 (2)	C8'—C7'—C6'—C5'	1.8 (3)
C9—C10—C11—N1	0.0 (3)	C7—C6—C5—C4	0.3 (3)
C1'—O2'—C2'—C3'	−170.42 (13)	C3—C4—C5—C6	−1.3 (3)

### Hydrogen-bond geometry (Å, °)

**Table:**

*D*—H···*A*	*D*—H	H···*A*	*D*···*A*	*D*—H···*A*
N2'—H2'C···N1^i^	0.86	2.09	2.9460 (16)	171
N2—H2C···N1'^ii^	0.86	2.11	2.9630 (18)	170

Symmetry codes: (i) −*x*+1, −*y*, −*z*; (ii) −*x*, −*y*, −*z*.
